# Graphene quantum dots induce cascadic apoptosis *via* interaction with proteins associated with anti-oxidation after endocytosis by *Trypanosoma brucei*

**DOI:** 10.3389/fimmu.2022.1022050

**Published:** 2022-12-06

**Authors:** Yiwei Xie, Hongrui Liang, Ning Jiang, Dingyuan Liu, Naiwen Zhang, Qilong Li, Kai Zhang, Xiaoyu Sang, Ying Feng, Ran Chen, Yiwei Zhang, Qijun Chen

**Affiliations:** ^1^ Key Laboratory of Livestock Infectious Diseases, Ministry of Education, Key Laboratory of Zoonosis, College of Animal Science and Veterinary Medicine, Shenyang Agricultural University, Shenyang, China; ^2^ Key Laboratory of Ruminant Infectious Disease Prevention and Control (East), Ministry of Agriculture and Rural Affairs, Shenyang, China; ^3^ Research Unit for Pathogenic Mechanism of Zoonotic Parasites, Chinese Academy of Medical Sciences, Shenyang Agricultural University, Shenyang, China

**Keywords:** graphene quantum dots, endocytosis, apoptosis, trypanothione reductase, anti-trypanosomials, *Trypanosoma brucei*

## Abstract

*Trypanosoma brucei*, the pathogen causing African sleeping sickness (trypanosomiasis) in humans, causes debilitating diseases in many regions of the world, but mainly in African countries with tropical and subtropical climates. Enormous efforts have been devoted to controlling trypanosomiasis, including expanding vector control programs, searching for novel anti-trypanosomial agents, and developing vaccines, but with limited success. In this study, we systematically investigated the effect of graphene quantum dots (GQDs) on trypanosomal parasites and their underlying mechanisms. Ultrasmall-sized GQDs can be efficiently endocytosed by *T. brucei* and with no toxicity to mammalian-derived cells, triggering a cascade of apoptotic reactions, including mitochondrial disorder, intracellular reactive oxygen species (ROS) elevation, Ca^2+^ accumulation, DNA fragmentation, adenosine triphosphate (ATP) synthesis impairment, and cell cycle arrest. All of these were caused by the direct interaction between GQDs and the proteins associated with cell apoptosis and anti-oxidation responses, such as trypanothione reductase (TryR), a key protein in anti-oxidation. GQDs specifically inhibited the enzymatic activity of TryR, leading to a reduction in the antioxidant capacity and, ultimately, parasite apoptotic death. These data, for the first time, provide a basis for the exploration of GQDs in the development of anti-trypanosomials.

## Introduction

African trypanosomiases are protozoan infectious diseases that mostly affect tropical and subtropical locations and pose a variety of health risks to humans and animals (1, 2). Some forms of African sleeping sickness are 100% fatal if not detected and treated. This condition threatens millions of people in 36 countries in Sub-Saharan Africa (3, 4). Vector control and prompt chemotherapy in infected humans and animals are the major means of disease control (5–7). Fexinidazole (8), an effective oral treatment for the first stage and the non-severe second stage of human African trypanosomiasis (HAT), should be administered for 10 days under the supervision of trained medical staff. Other drugs currently available for the treatment of trypanosomiasis in humans and animals are obsolete and have side effects. Suramin or pentamidine used for HAT must be administered by injection for a prolonged period, and both carry the risk of adverse events. Melarsoprol, which may cause an encephalopathic syndrome that is fatal in up to 1 in 20 individuals taking the drug, remains the treatment of choice for *Trypanosoma brucei rhodesiense* HAT (9, 10). Moreover, the emergence of drug resistance presents an obstacle to disease control (11, 12).

Research on trypanosomal vaccines has also faced great challenges due to the unlimited antigenic variation of the parasites (3). The lack of effective anti-trypanosomal medicines, along with failed vaccine development attempts, has sparked a flurry of research aimed at developing novel techniques and candidate pharmaceuticals to combat this disease.

Apoptosis is a type of cell death in which cells are killed by a sequence of events that do not encourage or provoke inflammatory responses (13, 14). Although it was once thought to be a phenomenon commonly occurring in multicellular organisms, new evidence suggests that it is a trait that also occurs in unicellular eukaryotes, including *Trypanosoma cruzi*, *T. brucei*, and *Leishmania* species (15–17). Apoptosis in trypanosomes has been suggested to improve biological fitness, in addition to modulating parasite density and the host immune system (18). Alternatively, approaches to promote or accelerate parasite apoptotic death are attractive for the development of novel anti-trypanosome medications.

Nanomaterials are increasingly being explored in biomedical applications owing to their nanoscale size, which provides unique and exceptional features (19–21). For example, silver (AgNPs) and gold nanoparticles (AgNPs) have been demonstrated to have antimicrobial and antiparasitic properties (22–24). The bioactivity of these nanoparticles, including selective binding and enzyme inhibition, has been reported in several studies (25, 26). However, extensive exploration remains pivotal for improving the clinical application of nanoparticle-based drugs.

In recent years, graphene-based nanoparticles have been employed as biomedical materials for the delivery of medical materials in several studies (27–29). Their unique electrochemical and mechanical properties, which have been explored for enzyme targeting, as well as the range of functional groups that may be modified on the surface, make these nanoparticles popular in medical nanomaterial development (30, 31). Graphene quantum dots (GQDs), a type of nanomaterial with comparable size to biomolecules, are less poisonous and hydrophobic than graphene and possess sustained strong photoluminescence to facilitate cell tracking after administration (32). Additionally, GQDs have a much faster renal clearance and biodegradation rate due to their small size. Their unique optical, electrochemical, and physicochemical properties enable many theranostic applications. Recently, GQDs have been proven to improve the chemotherapeutic efficacy of anticancer medications with less drug resistance due to their unique structural features (33).

Although GQDs have been widely investigated in biomedicine, there have been no reports on their use in trypanosomes. In this study, GQDs with a particle size of roughly 4.23 nm were generated, which sufficiently induced apoptosis in *T. brucei* with classical characteristics, including increased intracellular reactive oxygen radicals and Ca^2+^ concentration, DNA fragmentation, and decreased mitochondrial membrane potential (MMP) and adenosine triphosphate (ATP) production. We further observed that GQDs specifically interacted with and depressed the activity of trypanothione reductase (TryR), a key anti-oxidative stress protein in trypanosomal parasites.

## Materials and methods

### Materials

Carbon nanofibers (C139875-5G) were obtained from Aladdin (Shanghai, China). Fetal bovine serum (10099141C) and Iscove’s modified Dulbecco’s medium (IMDM; 12200036) were purchased from Gibco (Life Technologies, Gaithersburg, MD, USA). Trypsin–EDTA (MG0170) and penicillin–streptomycin (MG7989) were purchased from MacGene Biotechnology (Beijing, China). Nitric acid (225711), sulfuric acid (339741), sodium bicarbonate (S5761), thymidine (T1895), bathocuproine disulfonic acid disodium salt (B1125), l-cysteine (168149), and sodium pyruvate (P5280) were purchased from Sigma-Aldrich (St. Louis, MO, USA). Hypoxanthine (IH0490), dialysis membrane (YA1036) and Nuclear Extraction Kit (SN0020) were provided by Solarbio Life Science (Beijing, China). PrestoBlue cell viability reagents (A13261) were purchased from Invitrogen (Carlsbad, CA, USA). The annexin V-FITC Apoptosis Detection Kit (C1062L), Cell Cycle and Apoptosis Analysis Kit (C1052), Cell Counting Kit-8 (C0038), and one-step TUNEL (terminal deoxynucleotidyl transferase dUTP nick-end labeling) Apoptosis Assay Kit (C1088) were provided by Beyotime Biotechnology (Suzhou, China). The MMP assay kit (BB-3101), the intracellular Ca^2+^ assay kit (BB-48112), and the reactive oxygen assay kit (BB-47052) were provided by BestBio (Shanghai, China), while the ATP assay kit (A095-2-1) was provided by Jiancheng Bioengineering Institute (Nanjing, China). The HepG2 cell line was purchased from Cell Resource Center (Beijing, China). The *T. brucei* Lister strain 427 was maintained in our laboratory.

### Graphene quantum dots preparation and characterization

The GQDs were synthesized based on previous reports, with some modifications (34). Briefly, concentrated H_2_SO_4_ (60 ml) and HNO_3_ (20 ml) were mixed with 0.3 g of carbon nanofibers. The mixture was ultrasonically stirred for 2 h and further stirred at 100°C for 24 h and the GQDs generated. After cooling, the solution containing the GQDs was diluted with 600 ml Milli-Q water. Sodium carbonate was used to adjust the pH to 8. The GQDs were further purified by dialysis with a molecular weight cutoff of 2,000 Da. The particle sizes of the GQDs were characterized using transmission electron microscopy (Hitachi HT7800, Tokyo, Japan). The fluorescence spectra of the GQDs were recorded using a fluorescence spectrophotometer (Hitachi F-7100, Tokyo, Japan).

### Cell culture

The *T. brucei* Lister strain 427 was grown in an incubator at 37°C with 5% CO_2_ in IMDM supplemented with 10% fetal bovine serum (FBS) and 1% penicillin-streptomycin (35). Cell culture dishes (25 cm^2^) and 96-well plates were used for the experiments.

### Endocytosis of graphene quantum dots by *T. brucei*

*T. brucei* parasites (1 × 10^6^ cells/ml) were plated with a series of GQDs concentrations (0–400 μg/ml). After cultivation at 37°C and 5% CO_2_ for 6 h in a humidified incubator, *T. brucei* parasites were harvested and washed three times with phosphate-buffered saline (PBS). The endocytosis of GQDs particles in *T. brucei* was examined in comparison with parasites that were not incubated with GQDs using flow cytometry (BD Biosciences, San Jose, CA, USA) and a laser scanning confocal microscope (TCS SP8; Leica, Wetzlar, Germany) with fluorescence excitation/emission (*E*_x_/*E*_m_) at 360/440 nm.

### Clathrin-dependent endocytosis pathway in *T. brucei*

Inhibitors [Dynasore (36) and Pitstop2™ (37)] were used to investigate the clathrin-mediated endocytosis pathway. The parasites were grown in 96-well microplates (1 × 10^6^ cells/well) and pre-incubated for 30 min at 37°C with 10 and 20 μM Dynasore or 5 and 10 μM Pitstop2™. The parasites were maintained with 50 μg/ml GQDs for 3 h with 1 μM Dynasore or 0.5 μM Pitstop2™. To eliminate surface-adsorbed nanomaterials, *T. brucei* parasites were rinsed three times with PBS. The effect of Dynasore and Pitstop2™ on the endocytosis of GQDs by *T. brucei* was analyzed using flow cytometry and confocal microscopy.

### Cell viability and proliferation assays

The cell viability and proliferation after GQDs treatment were measured using the PrestoBlue™ (A13261; Invitrogen, Carlsbad, CA, USA) assay (38), which is a ready‐to‐use reagent for the rapid evaluation of the viability and proliferation of a wide range of cell types. In detail, viable cells continuously convert resazurin to highly fluorescent red resorufin, with increased overall fluorescence, and the cell viability can be detected using absorbance-based plate readers. *T. brucei* parasites (1 × 10^6^ cells/ml) were plated with varying concentrations of GQDs (0–400 μg/ml). After cultivation in a humidified incubator at 37°C with 5% CO_2_ for 12 and 24 h, 20 µl of the PrestoBlue™ Cell Viability Reagent solution was added to the samples. Thereafter, an additional 10 min of incubation was carried out at 37°C. The cells were then examined using a microplate reader (PerkinElmer, Billerica, MA, USA) with *E*_x_/*E*_m_ at 530/590 nm. *T. brucei* parasites that were not exposed to GQDs were used as controls.

Growth curves were determined by counting the parasites under a light microscope after being treated with GQDs at different time points. The abnormal nucleus and the kinetoplast morphology after GQDs treatment were calculated with a fluorescence microscope (39, 40). Parasite cells with one kinetoplast with one nucleus (1K1N), two kinetoplasts with one nucleus (2K1N), and two kinetoplasts with two nuclei (2K2N) were considered as normal cells, while parasites with 1K2N and *x*K*y*N (cells with multiple nuclei and kinetoplasts) were considered as abnormal cells.

### Apoptosis assays

Apoptosis was detected using the annexin V-FITC apoptosis detection kit (41). Briefly, *T. brucei* (1 × 10^6^ cells/ml) was plated with a series of GQDs concentrations (0–400 μg/ml). After cultivation in a humidified incubator at 37°C with 5% CO_2_ for 12 and 24 h, apoptotic *T. brucei* cells, in comparison with parasites that were not exposed to GQDs, were examined according to the manufacturer’s instructions and analyzed using flow cytometry (BD Biosciences, San Jose, CA, USA).

### Cell cycle assays

Cell cycle assays were performed using Cell Cycle Analysis Kit (42). *T. brucei* (1 × 10^6^ cells/ml) was exposed to GQDs (0–400 μg/ml) for 12 and 24 h before being harvested and washed three times with PBS. The samples were fixed in 70% cold ethanol and incubated for 12 h at 4°C. After fixation, the cells were resuspended in cold PBS to remove ethanol. The cells were then stained with propidium iodide (PI) (1 mg/ml) containing 10 mg/ml RNase for 30 min at 37°C. The cell cycle was assessed using flow cytometry (BD Biosciences, San Jose, CA, USA). *T. brucei* parasites that were not exposed to GQDs were used as controls.

### Assessment of intracellular ROS levels after exposure to GQDs

The intracellular reactive oxidative species (ROS) in *T*. *brucei* after treatment with GQDs were measured using a reactive oxygen detection kit (DHE-ROS) (43). *T. brucei* parasites (1 × 10^6^ cells/ml) were exposed to GQDs (0–400 μg/ml) for 12 and 24 h, and the samples were treated according to the manufacturer’s instructions and examined by flow cytometry at 488 nm excitation and 610 nm emission wavelengths (BD Biosciences, San Jose, CA, USA). *T. brucei* parasites cultivated simultaneously without exposure to GQDs were used as controls.

### Assessment of intracellular ATP after treatment with GQDs

The ATP levels in *T. brucei* after treatment with GQDs were measured using a firefly luciferase assay, which catalyzes the generation of luciferin in the presence of ATP (44). Briefly, *T. brucei* parasites (1 × 10^6^ cells/ml) were cultivated with varying doses of GQDs (0–400 μg/ml) for 12 and 24 h before the they were harvested. After lysis, the chemiluminescence of the supernatant was monitored using a microplate reader (PerkinElmer, Billerica, MA, USA). *T. brucei* parasites not incubated with GQDs were used as controls.

### Assessment of the mitochondrial membrane potential after GQDs treatment

*T. brucei* parasites (1 × 10^6^ cells/ml) were exposed to GQDs (0–400 μg/ml) for 12 and 24 h. Pathophysiological changes in the mitochondrial membrane potential (MMP) were measured using a JC-10 fluorescent probe, which accumulates in the matrix of mitochondria at high MMP and forms polymers emitting red fluorescence. On the contrary, at low MMP, JC-10 forms monomers emitting green fluorescence in the matrix of the mitochondria. The fluorescence of JC-10 aggregates and JC-10 monomers was detected by flow cytometry with an excitation wavelength of 488 nm and emission wavelengths of 570 and 520 nm, respectively (45). *T. brucei* parasites that were not exposed to GQDs were used as controls.

### Measurement of DNA fragmentation after GQDs treatment

*T. brucei* DNA fragmentation after treatment with GQDs was analyzed using a TUNEL assay, which uses terminal deoxynucleotidyl transferase to add fluorescently labeled dUTP to the 3′-OH ends of the DNA fragments, which resulted from the apoptotic process (46). *T. brucei* parasites (1 × 10^6^ cells/ml) were cultivated with varying doses of GQDs (0–400 μg/ml) for 12 and 24 h before the they were harvested. Apoptotic cells with DNA fragmentation were detected and analyzed using flow cytometry with *E*_x_/*E*_m_ at 475/520 nm. *T. brucei* parasites that were not exposed to GQDs were used as controls.

### Assessment of intracellular Ca^2+^ of *T. brucei* after GQDs treatment

Cytosolic Ca^2+^ concentrations were measured using BBcellProne^®^ F03 (BB-48112; BestBio Technical, Beijing, China) according to the manufacturer’s instructions (47). *T. brucei* parasites (1 × 10^6^ cells/ml) were cultivated using a variety of GQDs doses (0–400 μg/ml) for 12 and 24 h. Thereafter, the cells were collected and washed three times in PBS. Subsequently, the parasites were treated with the BBcellProne^®^ F03 staining working solution at 37°C for 30 min, washed three times with PBS, and cultivated for a further 30 min in the culture medium. The fluorescence intensity of the parasites was obtained by obtaining the emission at 488 nm using flow cytometry (BD Biosciences, San Jose, CA, USA) and further examining with a laser scanning confocal microscope (TCS SP8; Leica, Wetzlar, Germany). *T. brucei* parasites not incubated with GQDs were used as controls.

### Western blotting analysis of tSNAP42 and Endo G in *T. brucei* nuclei after GQDs treatment

The nuclei of *T. brucei* cells were isolated using a nuclear extraction kit (SN0020; Solarbio, Beijing, China) following the manufacturer’s instructions. The 1× SDS-PAGE loading buffer was mixed with the samples before being heated at 100°C for 5 min. Afterward, the proteins were separated by 12% SDS-PAGE and transferred to polyvinylidene difluoride (PVDF) membranes. Before co-incubation with rabbit anti-tSNAP42 immunoglobulin G (IgG) (1:1,000), rabbit anti-endonuclease G (Endo G) IgG (1:1,000), and rabbit anti-histone 3 IgG (1:1,000), the membranes were blocked with 5% skim milk at 37°C for 1 h. Horseradish peroxidase (HRP)-conjugated goat anti-rabbit IgG (SE134; Solarbio, Beijing, China) was used as the secondary antibody. The nuclear proteins of *T. brucei* parasites not exposed to GQDs were used as controls.

### Flow cytometry analysis of tSNAP42 and Endo G in *T. brucei* after GQDs treatment

A glutaraldehyde solution (2.5%) was used to fix *T. brucei* parasites treated with GQDs. After washing with PBS, the cells were blocked with 5% (*w*/*v*) skim milk. Rabbit anti-tSNAP42 IgG and rabbit anti-Endo G IgG were added to the cells. Alexa Fluor 488-conjugated goat anti-rabbit IgG was used as the secondary antibody. Finally, the fluorescence intensity of the parasites was determined by measuring the emission at 488 nm using flow cytometry (BD Biosciences, San Jose, CA, USA). *T. brucei* parasites that were not exposed to GQDs were used as controls.

### Transcriptomic analysis on *T. brucei* after GQDs treatment

After exposure to GQDs (50 μg/ml) for 24 h, *T. brucei* parasites (1 × 10^7^ cells/ml) were dissolved in TRIzol reagent for the extraction of total RNA. The enrichment of messenger RNA (mRNA) was carried out with Oligo(dT) beads and the Ribo-Zero™ Magnetic Kit (MRZH11124; Epicenter Biotechnologies, Madison, WI, USA). RNA sequencing (RNA-seq) was performed by Gene Denovo Biotechnology (Guangzhou, China) using Illumina HiSeq2500.

Differential transcription analysis of mRNA was conducted using DESeq2 software (48) and edgeR (49) between the sequences of GQD-treated and untreated parasites. A false discovery rate (FDR) ≤0.05 and fold change ≥1.5 were used as the thresholds for differentially expressed genes (DEGs). Proteins encoded by DEGs were subjected to Gene Ontology (GO) (50) and Kyoto Encyclopedia of Genes and Genomes (KEGG) pathway analyses (51). Significantly enriched GO terms or pathways were defined as FDR ≤ 0.05.

### Confirmation of trypanothione synthetase and trypanothione reductase transcription by quantitative real-time PCR

After exposure to GQDs (50 μg/ml) for 24 h, *T. brucei* parasites (1 × 10^7^ cells/ml) were dissolved in TRIzol reagent for the extraction of total RNA. SYBR^®^ Premix Ex Taq™ (RR820A; TaKaRa, Shiga, Japan) was used for real-time PCR. The specific primers corresponding to the genes coding for both trypanothione synthetase and TryR are listed in **Supplementary Table S1**. The gene transcription level was determined using the 2^−ΔCt^ method, which compares the quantity of the target RNA to that of the gene encoding glyceraldehyde-3-phosphate dehydrogenase (*GAPDH*; Tb927.6.4280), which was used as an internal control (52).

### Localization with immunofluorescence and expression analysis of the trypanothione reductase of *T. brucei* after GQDs treatment

Thin smears of *T. brucei* were fixed in cold methanol for 10 min before blocking in 5% skim milk. The mouse anti-TryR antibody was used as the primary antibody at a dilution of 1:50. Alexa Fluor 594-conjugated goat anti-mouse IgG (SE134; Solarbio, Beijing, China) was used as the secondary antibody at a dilution of 1:600. The nuclei of the parasites were stained with DAPI. *T. brucei* parasites that were not exposed to GQDs were used as controls. Serum from an unimmunized mouse and PBS buffer without antibodies were used as the negative and blank controls, respectively. High-resolution images were captured using a confocal laser scanning microscope (SP8; Leica, Wetzlar, Germany).

The total lysate of *T. brucei* that received GQDs treatment was mixed with the SDS-PAGE loading buffer, and the proteins were separated using 12% SDS-PAGE. Subsequently, the proteins were transferred onto PVDF membranes. Before co-incubation with mouse anti-TryR IgG (1:1,000), the membranes were blocked with 5% skim milk at 37°C for 1 h. HRP-conjugated goat anti-rabbit IgG (SE134; Solarbio, Beijing, China) was used as the secondary antibody. Signals obtained with rabbit anti-GAPDH IgG were used for normalization.

### Kinetic analysis of the interaction between GQDs and trypanothione reductase

The gene coding for the TryR of *T. brucei* (Tb927.10.10390) was amplified from parasite complementary DNA (cDNA) and cloned into the expression vector pET-28a in *Escherichia coli* BL21 (DE3) cells. The His-tagged recombinant protein (TryR-His) was purified using NiNTA agarose (Qiagen, Germantown, MD, USA) according to the manufacturer’s instructions.

The interaction kinetics between the GQDs and TryR-His were studied using an Octet K2 device (ForteBio, Menlo Park, CA, USA). Firstly, the Ni-NTA biosensors were coated with TryR-His (50 μg/ml). GQDs at a series of concentrations were then loaded onto the Ni-NTA biosensors. Subsequently, the binding affinities of the GQDs (diluted from 800 to 12.5 nM) with TryR-His were determined. The association and dissociation between GQDs and TryR-His were monitored for 5 min, and Octet Data Analysis software (version 7.0; ForteBio, Menlo Park, CA, USA) (53) was used to calculate the affinities and kinetic parameters.

### Statistical analysis

All experiments were performed in triplicate. Student’s *t*-test was used to analyze the data. All values are presented as the mean ± SD of triplicate measurements. *p < 0.05 was considered significant, **p < 0.01 considered highly significant, and ***p < 0.001 was considered very highly significant.

## Results and discussion

### Synthesis and characterization of graphene quantum dots

**Figure 1:**
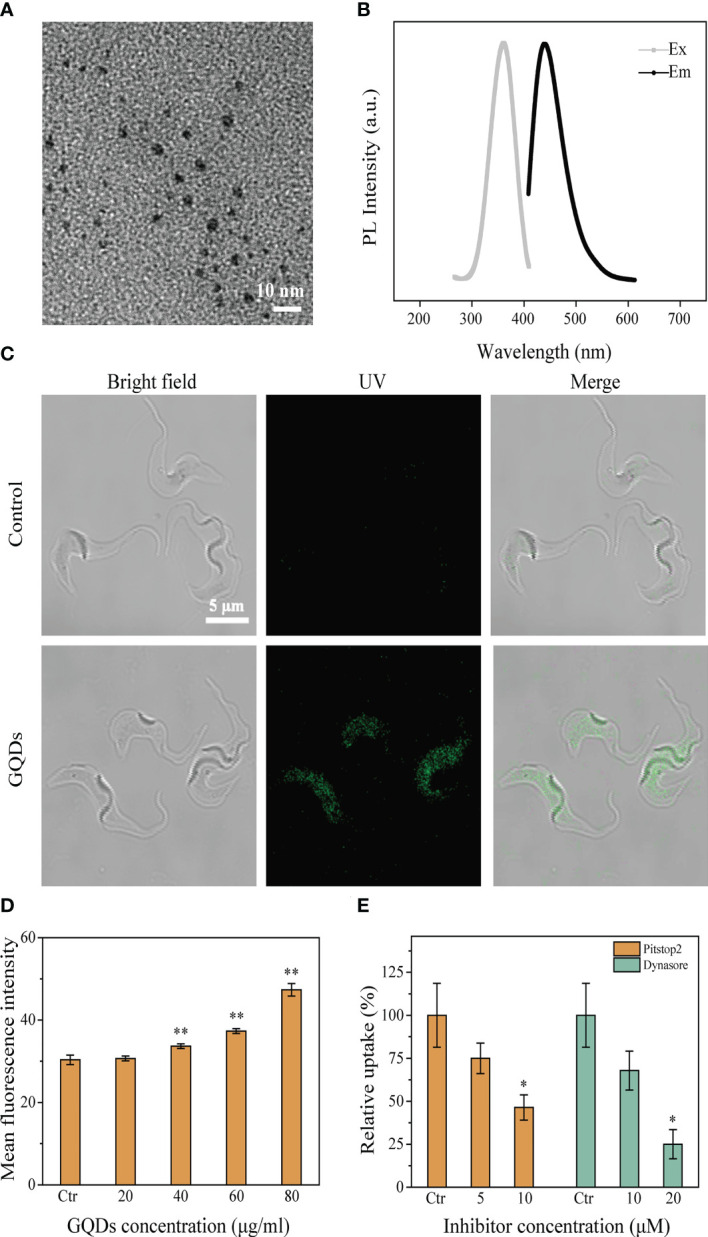
Characteristics of graphene quantum dots (GQDs) and their intracellular distribution in *Trypanosoma brucei* after co-incubation. **(A)** TEM images of GQDs (×80K). **(B)** Fluorescence spectra of GQDs. **(C)** Distribution of GQDs in *T. brucei* after incubation for 3 h. *T. brucei* with untreated parasites was taken as the control. GQDs were endocytosed and mainly distributed in the cytosol of the parasites. **(D)** Flow cytometry was used to measure the intracellular fluorescence intensity after incubation for 3 h with a series of concentrations of GQDs. The fluorescence intensified with the increase in the concentration of GQDs concentration. **(E)** Clathrin inhibitors (Pitstop2™ and Dynasore) impeded the endocytosis of GQDs by *T. brucei.* The inhibitory effect steadily increased with the increase in inhibitor concentration, measured by flow cytometry after 3 h of treatment. *T. brucei* parasites cultivated with GQDs without inhibitors were used as controls. All values are the mean ± SD of triplicates. *p < 0.05, **p < 0.01.

The synthesized GQDs were morphologically characterized using a transmission electron microscope (TEM) (**Figure 1A**). The size distribution of the GQDs was 4.23 ± 0.94 nm (*n* = 100) (**Supplementary Figure S1**). The GQDs were scanned using a fluorescence spectrophotometer to obtain the fluorescence spectra (**Figure 1B**). Based on the fluorescence spectrum, an excitation wavelength of 360 nm and an emission wavelength of 440 nm were chosen to analyze the endocytosis of GQDs by *T. brucei*.

### *Trypanosoma brucei* endocytosed GQDs *via* the clathrin-dependent pathway

The entry of GQDs into *T. brucei* was observed using a laser scanning confocal microscope. After co-incubation with *T. brucei* cells, a wide distribution of GQDs in the cytoplasm of the parasites was observed (**Figure 1C**), and the quantity of intracellular GQDs was concentration-dependent (**Figure 1D**). Considering that trypanosome parasites utilize the clathrin-mediated endocytosis pathway (54), two well-known clathrin inhibitors (Pitstop2™ and Dynasore) were selected for the analysis of the transportation of GQDs into *T. brucei*. The uptake of GQDs by *T. brucei* was considerably reduced in a concentration-dependent manner when these inhibitors were used (**Figure 1E**). Prior to the experiment, the toxicity of the two inhibitors to parasites was determined using a Cell Counting Kit-8 assay. Both Pitstop2™ and Dynasore did not show cytotoxicity to parasites at the applied concentrations (**Supplementary Figure S2**). This finding suggests that *T. brucei* endocytosed GQDs *via* a clathrin-dependent pathway.

### GQDs were cytotoxic to *T. brucei* and could efficiently induce parasite apoptosis

**Figure 2:**
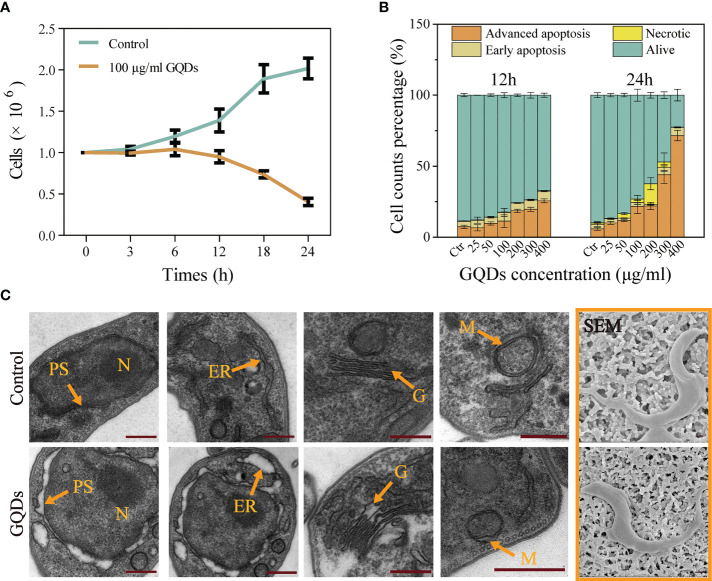
Growth curves and subcellular organelle pathophysiological changes in *Trypanosoma brucei* following treatment with graphene quantum dots (GQDs). **(A)** Growth curves based on nuclear counts of *T. brucei* after treatment with GQDs for 24 h. *T. brucei* that did not receive GQDs treatment were used as controls. **(B)** Apoptosis of *T. brucei* induced by GQDs at 12 and 24 h. The parasites primarily displayed advanced apoptosis after treatment. Statistical studies were performed based on the flow cytometry data in **Supplementary Figure S6**. Early apoptosis was manifested as green fluorescence in combination with annexin V-FITC. Necrosis was manifested as no change in membrane permeability, while nuclei showed red fluorescence in combination with the fluorescent dye propidium iodide. Cells stained with two dyes were considered to be in the advanced apoptosis stage. **(C)** TEM and SEM images of *T. brucei* captured after cultivation with GQDs (50 μg/ml) for 24 h. *Orange arrows* indicate organelles. *N*, nuclei; *ER*, endoplasmic reticulum; *G*, Golgi apparatus; *M*, mitochondria; *PS*, perinuclear space. *T. brucei* parasites that were not exposed to GQDs were taken as controls. *Scale bar*, 500 nm. All values are the mean ± SD of triplicates. Student’s *t*-test was used to calculate the *p*-values. *p < 0.05, **p < 0.01, ***p < 0.001 (compared to the control).

The cytotoxicity of GQDs is believed to be closely associated with their size, charge, the biological activity (functional groups) of the outer layer, oxidation, photolysis, and mechanical stability (55, 56). Although studies have demonstrated that ultra-small nanomaterials are less cytotoxic than large-sized nanomaterials (>100 nm), the ultra-small GQDs we generated displayed outstanding cytotoxicity to *T. brucei*. Treatment with GQDs decreased the cell proliferation based on the proliferation curve (**Figure 2A**) and the arrest of cell development (**Supplementary Figure S3**) of *T. brucei*, and the half maximal effective concentration (EC_50_) for GQDs at 24 h was 27.16 μg/ml. Based on the assessment of HepG2 cells (**Supplementary Figure S4**) and on previous reports (57), the doses of the GQDs explored in this study were not toxic to mammalian cells. The annexin V-FITC/PI apoptosis assay (58) was used to assess the apoptosis of *T. brucei* cells following treatment with GQDs. GQDs mostly promoted advanced apoptosis in *T. brucei* (**Figure 2B**).

Although the morphological characteristics of *T. brucei* were not altered significantly (**Figure 2C** and **Supplementary Figure S5**, SEM images), a series of classical cell apoptosis features with subcellular morphological defects were observed after exposure to GQDs, similar to the cytopathological alterations caused by other nanomaterials (59). The pathophysiological changes in *T. brucei* subcellular organelles (**Figure 2C**, TEM images) were dramatic following treatment with GQDs, including mitochondrial (M) morphological alterations and a swollen Golgi apparatus (G). Additionally, the perinuclear space (PS) was severely swollen after exposure to GQDs. Dilatation of the endoplasmic reticulum (ER) was also observed (60).

**Figure 3:**
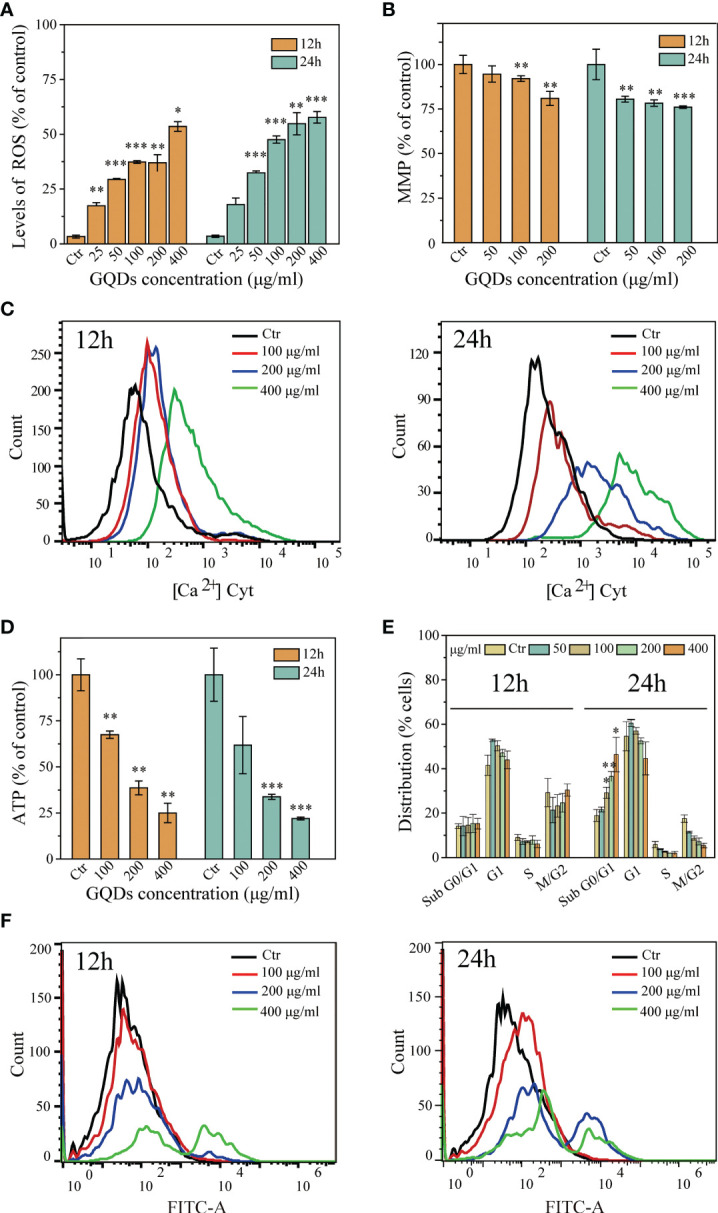
Cytotoxicological effects graphene quantum dots (GQDs) on *Trypanosoma brucei* 12 and 24 h post-exposure. **(A)** Quantitative analysis of *T. brucei* intracellular reactive oxygen species (ROS) values based on the results in **Supplementary Figure S7**. The intracellular ROS levels increased with prolonged exposure and increased doses. **(B)** Alterations of the MMP (Δ*Ψ*_m_) in *T. brucei* following treatment with GQDs. The levels of Δ*Ψ*_m_ decreased with increased doses and duration of exposure. A JC-10 fluorescent probe was applied for the detection using flow cytometry (**Supplementary Figure S8**). **(C)** Increased cytoplasmic Ca^2+^ concentration of *T. brucei* following treatment with GQDs for 12 and 24 h, demonstrated by flow cytometry. Changes in the intracytoplasmic Ca^2+^ concentration captured by confocal microscopy, which can be seen in **Supplementary Figure S9**. **(D)** Intracellular ATP was significantly decreased in *T. brucei* after exposure to GQDs. **(E)** Exposure to GQDs resulted in the developmental arrest of *T. brucei.* The proportions of cell cycle dispersion are shown in graphs, predicated in the flow cytometry data in **Supplementary Figure S10**. **(F)** Flow cytometry analysis of the increased DNA fragmentation in *T. brucei* after treatment with varying concentrations of GQDs for 12 and 24 h using TUNEL (terminal deoxynucleotidyl transferase dUTP nick-end labeling) staining. *T. brucei* parasites that were not exposed to GQDs served controls. All values are the mean ± SD of triplicates. Student’s *t*-test was used to obtain *p*-values. *p < 0.05, **p < 0.01, ***p < 0.001 (compared to the control).

An increase in ROS level is a key feature of nanoparticle-induced apoptosis in mammalian cells. The administration of GQDs led to a tremendous increase in the intracellular ROS levels, which was accompanied by a progressive reduction in the MMP (Δ*Ψ*_m_) in a dose-dependent manner (**Figures 3A, B** **)**.

The intracellular accumulation of free radicals and the increased Ca^2+^ levels can lead to MMP (Δ*Ψ*_m_) depression, further leading to cell apoptosis. We subsequently investigated alterations in the concentrations of Ca^2+^ in *T. brucei* after incubation with GQDs. Treatment with GQDs resulted in intracellular Ca^2+^ accumulation in *T. brucei* (**Figure 3C**). Furthermore, defects in the ATP generation have been regarded as a consequence of mitochondrial dysfunction and apoptosis. In this study, GQDs were also found to effectively suppress the ATP generation in *T. brucei* (**Figure 3D**), which was congruent with the loss of MMP and the elevation of the concentrations of intracellular ROS and Ca^2+^. Moreover, treatment with GQDs resulted in developmental arrest in the sub-G0/G1 phase of *T. brucei* cells and a concomitant decline in G2/M phase cells (**Figure 3E**), which is typically caused by DNA fragmentation. Considerable DNA fragmentation in *T. brucei* was also observed after 24 h of exposure to GQDs (**Figure 3F**). Considering that GQDs did not exhibit cytotoxic effects on the host cells, these data indicate that GQDs exerted specific biological inhibition on trypanosomal parasites.

### Pathways related to apoptosis in *T. brucei* induced by GQDs

**Figure 4:**
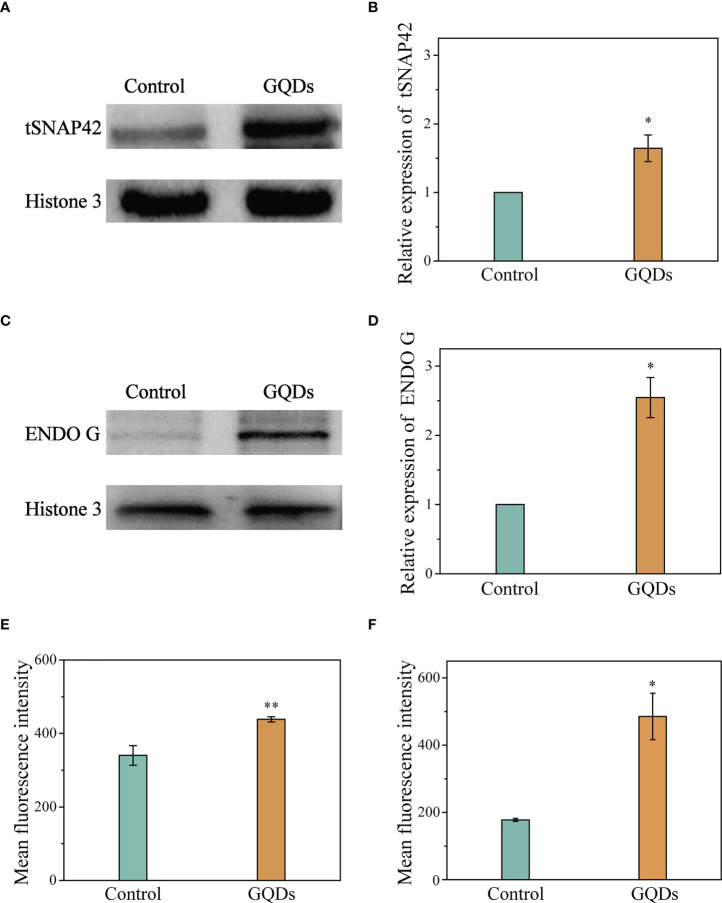
Elevation of the apoptosis-associated proteins of *Trypanosoma brucei* after exposure to graphene quantum dots (GQDs). **(A)** Increased intracellular accumulation of tSNAP42 in *T. brucei* after treatment with GQDs as revealed by Western blotting analysis. Histone 3 served as the control. **(B)** Densitometric analysis of the gray images in **(A)** using ImageJ software (v1.8.0). **(C)** Increased quantity of endonuclease G (Endo G) in *T. brucei* revealed by Western blotting analysis. Histone 3 served as the control. **(D)** Densitometric analysis of the gray images in **(C)** using ImageJ software (v1.8.0). **(E, F)** Increased intracellular accumulation of tSNAP42 **(E)** and Endo G **(F)** in *T. brucei* after treatment with GQDs as determined by flow cytometry (**Supplementary Figure S11**). *T. brucei* parasites that were not exposed to GQDs served as controls. Data shown are the mean ± SD (*n* ≥ 3). Student’s *t*-test was used to obtain *p*-values. *p < 0.05, **p < 0.01 (compared to the control).

In *T. brucei*, prolonged ER stress induces an apoptotic mechanism called splice leader RNA silencing (SLS), which resembles apoptosis mediated by caspases observed in eukaryotic organisms (61). The hallmark of SLS is the shut-off of the transcription of the splice leader RNA (SL RNA) and the massive accumulation of tSNAP42, a specific transcription factor that binds the promoter of SL RNA (62). In this study, exposure to GQDs caused a significant elevation of tSNAP42 in the cell nuclear fraction after prolonged ER stress, as revealed by Western blotting (**Figures 4A, B** **)**. Although trypanosomatids have an apoptotic mechanism independent of caspases, Endo G, a well-described nuclease released from the mitochondria and its role in DNA fragmentation following trafficking to the nucleus have been documented (63). Therefore, we used Western blotting to investigate organelle fractionation in *T. brucei* following exposure to GQDs and observed considerable accumulation of Endo G in the nuclear fractions (**Figures 4C, D** **)**. Flow cytometry studies also revealed that the intracellular expression levels of tSNAP42 and Endo G were remarkably increased following exposure to GQDs, suggesting that the mitochondrial pathway and ER stress were indeed pivotal in the apoptosis of *T. brucei* induced by GQDs (**Figures 4E, F** **)**.

### Alterations in the gene expression of *T. brucei* were tightly correlated to GQDs treatment

**Figure 5:**
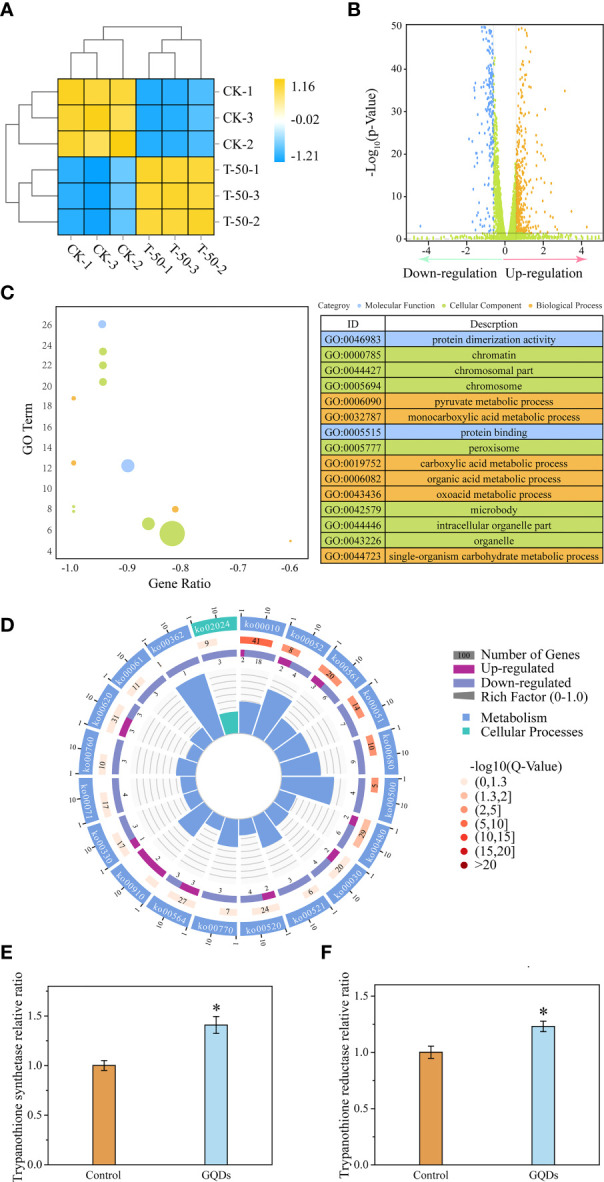
Transcriptomic analysis and key genes related to the redox function of *Trypanosoma brucei* after exposure to graphene quantum dots (GQDs). **(A)** Correlation analysis of the transcriptomic data between GQDs-treated parasites and controls following treatment in *T. brucei*. *CK-1*, *CK-2*, and *CK-3* represent triplicate samples without GQDs treatment. *T-50-1*, *T-50-2*, and *T-50-3* represent triplicate samples treated with GQDs (50 μg/ml). *Orange* indicates positive correlation, while *blue* denotes negative correlation. **(B)** Distribution of the differentially expressed genes (DEGs) after exposure to GQDs. For transcriptomic analysis, normalized data were *p* ≤ 0.05 (**Dataset S1**). *Brown color* represents upregulated genes, *blue* indicates downregulated genes, and *green* represents the genes without significant changes. A false discovery rate ≤0.05 and fold change ≥1.5 were used as the thresholds for DEGs. **(C)** Top 15 enriched Gene Ontology (GO) terms based on the DEGs following GQDs treatment in *T. brucei* (**Dataset S2**). **(D)** Corresponding pathways significantly enriched based on the DEGs after exposure to GQDs in *T. bruce*i. For transcriptomic analysis, normalized data were *p* ≤ 0.05 (**Dataset S3**). **(E, F)** Transcriptional analysis of the trypanothione synthase **(E)** and trypanothione reductase **(F)** of *T. brucei* following treatment with GQDs by quantitative reverse transcription PCR (qRT-PCR). The *GADPH* gene served as a standardization control. Values are the mean ± SD of triplicates. Student’s *t*-test was used to obtain *p*-values. *p < 0.05 (compared to the control).

To further investigate the molecular mechanisms of apoptosis in *T. brucei* induced by treatment with GQDs, parasite RNAs were sequenced after treatment (50 µg/ml) for 24 h. Among the 10,277 transcripts identified, 783 were found to be differentially transcribed, some of which were linked to mitochondrial function, oxidative stress, energy metabolism, glycometabolism, and lipid metabolism (**Figures 5A, B** **)**. The most upregulated gene was a gene coding for the variant surface glycoprotein (6.7, Tb927.3.5900). The expression levels of the GPEET2 procyclin precursor (3.1, Tb927.6.510), EP1 procyclin precursor (2.4, Tb927.10.10260), and the protein encoded by the expression site-associated gene 1 (*ESAG 1*) (3.5, Tb927.3.2520) were also highly upregulated. In addition, the upregulation of iron/ascorbate oxidoreductase (2.1, Tb927.2.6270) was closely related to the redox regulatory system. The gene with the most pronounced fold downregulation was adenylate cyclase (−4.4, Tb927.11.1490), which was most likely due to the reduction in ATP production (**Dataset S1**), indicating elevated response to the GQDs treatment.

We used GO and KEGG enrichment analyses with a corrected *p*-value of 0.05 as the threshold to acquire more thorough insights into the functions of the DEGs. Protein function, chromosome associates, peroxisomes, and pyruvate metabolism were among the top 10 enriched terms (**Figures 5C, D** **)**. Furthermore, genes involved in glycerolipid metabolism, which is closely related to lipid peroxidation, were significantly enriched. This also indicates that the antioxidant capacity of the parasites may have been profoundly impaired by GQDs treatment. Moreover, the genes involved in the glycolysis/gluconeogenesis and pentose phosphate pathways showed a significant enrichment, indicating that *T. brucei* may perform a correspondent adjustment in the energy metabolism under GQDs treatment (**Figure 5C**).

Trypanosomal parasites do not contain the conventional disulfide reductases (glutathione reductase and thioredoxin reductase) seen in higher eukaryotes and have developed a distinctive redox system relying on trypanothione, which is considered to be a protective mechanism that maintains intracellular redox homeostasis (64). Quantitative reverse transcription PCR (qRT-PCR) (**Figure 5E**) and RNA-seq (**Supplementary Figure S12A**) revealed the enhanced expression of trypanothione synthetase in response to GQDs treatment, indicating that GQDs induced oxidative stress in parasites. However, the expression of TryR was slightly suppressed in the transcriptomic analysis (**Supplementary Figures S12B** and **S13**), which was different from the result of qRT-PCR (**Figure 5F**). This was likely caused by intrinsic deficiency of the RNA-seq approach (65–67). Multiple qRT-PCR experiments showed that the expression of TryR was upregulated after treatment with GQDs.

### GQDs directly interacted with the trypanothione reductase of *T. brucei* and inhibited its enzymatic activity

**Figure 6:**
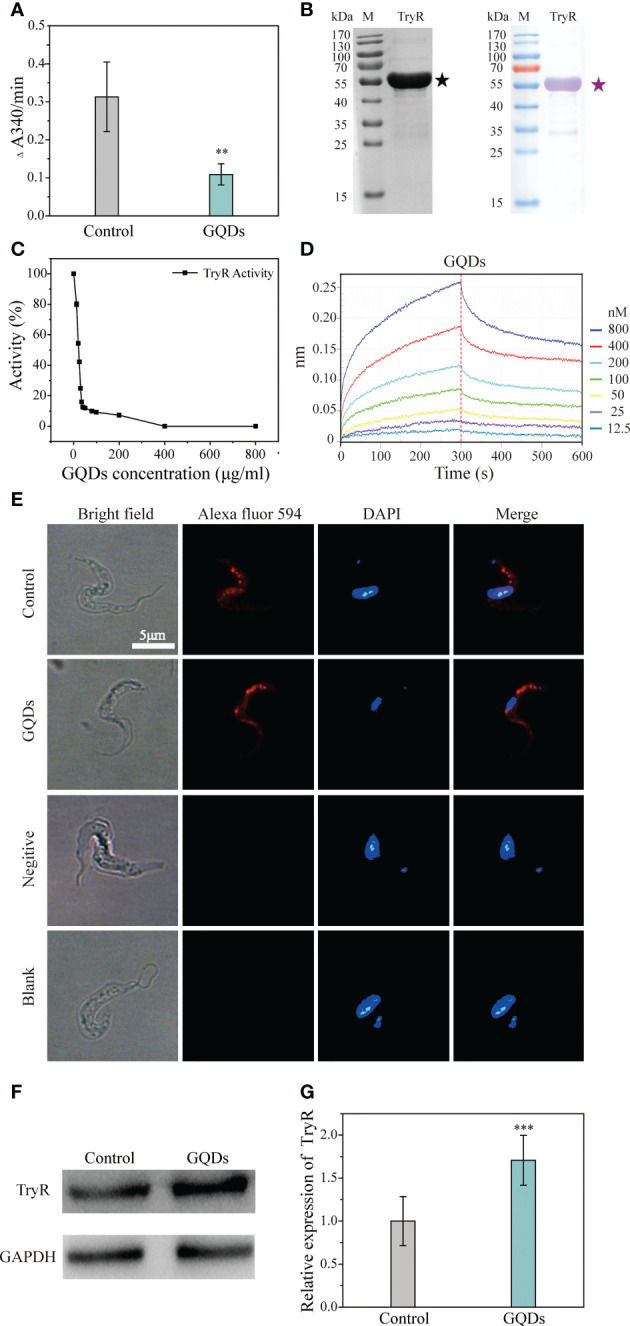
Direct affinity analysis and functional inhibition of the trypanothione reductase (TryR) of *Trypanosoma brucei* by graphene quantum dots (GQDs). **(A)** The catalytic activity of total parasite lysate was depressed in *T. brucei*, represented by the reduction of trypanothione after treatment with GQDs. The lysate of *T. brucei* not exposed to GQDs served as the control. **(B)** Analysis of the purified recombinant TryR protein using SDS-PAGE (*left*) and Western blotting (*right*). **(C)** GQDs treatment inhibited the catalytic activity of the recombinant TryR of *T. brucei* in a concentration-dependent manner. **(D)** Label-free binding of GQDs to the immobilized His-tagged recombinant TryR (TryR-His) in the Octet. To describe the intensity of the binding affinity between the two compounds, the equilibrium dissociation constant (*K*_d_) was calculated with *k*_dis_/*k*_on_. *K*_d_ values from 1.0E−03 to 1.0E−12 M indicate that two compounds are interacting (**Supplementary Table S2**). **(E)** Distribution of TryR in the parasites evaluated by immunofluorescence assay. A mouse anti-TryR IgG and Alexa Fluor 594-conjugated goat anti-mouse IgG were employed as the primary and secondary antibodies, respectively. Nuclei and kinetoplasts were stained with DAPI. The buffer without antibodies and unimmunized mouse serum were used as the blank and negative controls, respectively. *Cyan* indicates saturated pixels. **(F)** Increased intracellular accumulation of TryR in *T. brucei* after GQDs treatment as revealed by Western blotting analysis. *GADPH* was adopted as the control. **(G)** Densitometric analysis of the gray images in **(F)** using ImageJ software (v1.8.0). All values are the mean ± SD of triplicates. Student’s *t*-test was used to obtain *p*-values. ***p < 0.001 (compared to the control) (26).

**Figure 7:**
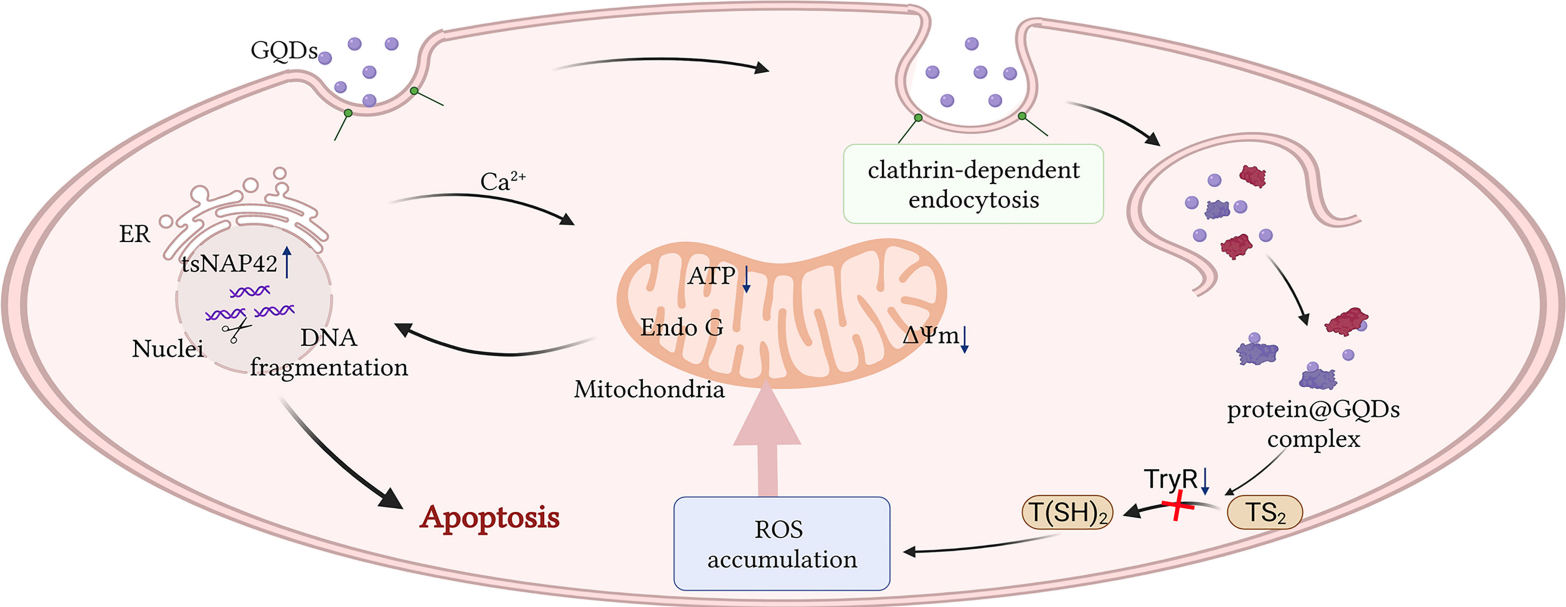
Schematic description of the apoptotic process caused by graphene quantum dots (GQDs) in *Trypanosoma brucei*. GQDs were internalized by *T. brucei* through clathrin-mediated endocytosis and interacted with several proteins, including trypanothione reductase (TryR), a key enzyme responsible for catalyzing trypanothione disulfide (TS_2_) to trypanothione [T(SH)_2_] in the anti-oxidation of trypanosome. The reduced TryR activity resulted in the accumulation of intracellular reactive oxygen species (ROS), eventually inducing apoptosis *via* mitochondrial and endoplasmic reticulum (ER) stress pathways. This figure was created by biorender. The agreement number is FP24OK0AS9.

Previous studies have reported that nanomaterials can be efficiently endocytosed, resulting in increased intracellular reactive oxygen levels through interactions with intracellular proteins and DNAs (55). Nanomaterials have also been shown to affect the secondary structure of proteins, which may cause changes in their functions (68). TryR is a pivotal enzyme in the response to oxidative stress in trypanosomal parasites, and its expression and activity have a substantial impact on the antioxidant capability. Cell physiological analysis revealed that the redox capability was impeded by GQDs treatment. To confirm this, a total lysate of *T. brucei* containing TryR was obtained for the test of antioxidant activity with trypanothione as the substrate. The antioxidant capacity of the lysate from parasites pre-exposed to GQDs was significantly lower than that of untreated parasites (**Figure 6A**). Recombinant TryR was obtained and confirmed using Western blotting analysis (**Figure 6B**). GQDs inhibited the activity of recombinant TryR (TryR-His) in a dose-dependent manner (**Figure 6C**). The affinity of GQDs to TryR-His was analyzed using the Octet K2 system, which tracked the binding (*k*_on_) and dissociation *k*_dis_) rates between the two molecules. The dissociation constant (*K*_d_) for the binding between TryR-His and GQDs was 5.04E−09. This suggests that GQDs have a high affinity for TryR (**Figure 6D** and **Supplementary Table S2**), which explains their dramatic antiparasitic effects. Furthermore, immunofluorescence indicated that the intracellular distribution of TryR was not affected by treatment with GQDs (**Figure 6E**). GQDs were not observed in the DAPI channel of the second row because they were below the detection threshold for this exposure time. Western blotting analysis showed that the expression of TryR also increased in response to GQDs treatment (**Figures 6F, G** **)**. These data indicate that the activity of TryR was inhibited after GQDs treatment, which ultimately resulted in the constant accumulation of ROS in the parasites and the onset of apoptosis (**Figures 3A** and **7**).

## Conclusion

In summary, our study revealed that GQDs are sufficiently endocytosed by *T. brucei*, which specifically impedes the redox system and triggers a cascade of apoptotic reactions in parasites. The data systematically revealed the molecular mechanism by which GQDs induce trypanosomal apoptosis, arguing for further exploration of the development of anti-trypanosomials based on GQDs materials.

## Data availability statement

The datasets presented in this study can be found in online repositories. The names of the repository/repositories and accession number(s) can be found in the article/**Supplementary material**.

## Author contributions

YX performed most of the experiment and wrote the first draft of the manuscript. HL performed most of the experiment. NJ mentored the study. DL performed the enzymatic experiments. NZ and KZ assisted in parasite cultivation and the viability assays. QL assisted in the bioinformatic analysis. XS assisted in the cell apoptosis assays. YF performed the confocal microscopy experiments. RC and YZ performed the flow cytometry experiments. QC conceived the study, analyzed the data, and finalized the manuscript. All authors contributed to the article and approved the submitted version.

## Funding

This study was supported by the National Natural Science Foundation of China (grant no. 32072880); and the CAMS Innovation Fund for Medical Sciences (2019-I2M-5-042).

## Acknowledgments

Technical assistance from the Department of Core Facility of Shenyang Agricultural University in the electromicroscopy experiments of the samples is very much appreciated. 

## Conflict of interest

The authors declare that the research was conducted in the absence of any commercial or financial relationships that could be construed as a potential conflict of interest.

## Publisher’s note

All claims expressed in this article are solely those of the authors and do not necessarily represent those of their affiliated organizations, or those of the publisher, the editors and the reviewers. Any product that may be evaluated in this article, or claim that may be made by its manufacturer, is not guaranteed or endorsed by the publisher.
