# Supplementary material for: Graphene quantum dots induce cascadic apoptosis via interaction with proteins associated with anti-oxidation after endocytosis by Trypanosoma brucei
Source: Front Immunol. 2022 Dec 6;13:1022050. doi: 10.3389/fimmu.2022.1022050 (PMC9763322; doi:10.3389/fimmu.2022.1022050)
Supplement: Supplementary file 1 [file DataSheet_1.docx]

***Supplementary materials***

**1. Supplemental Figures**


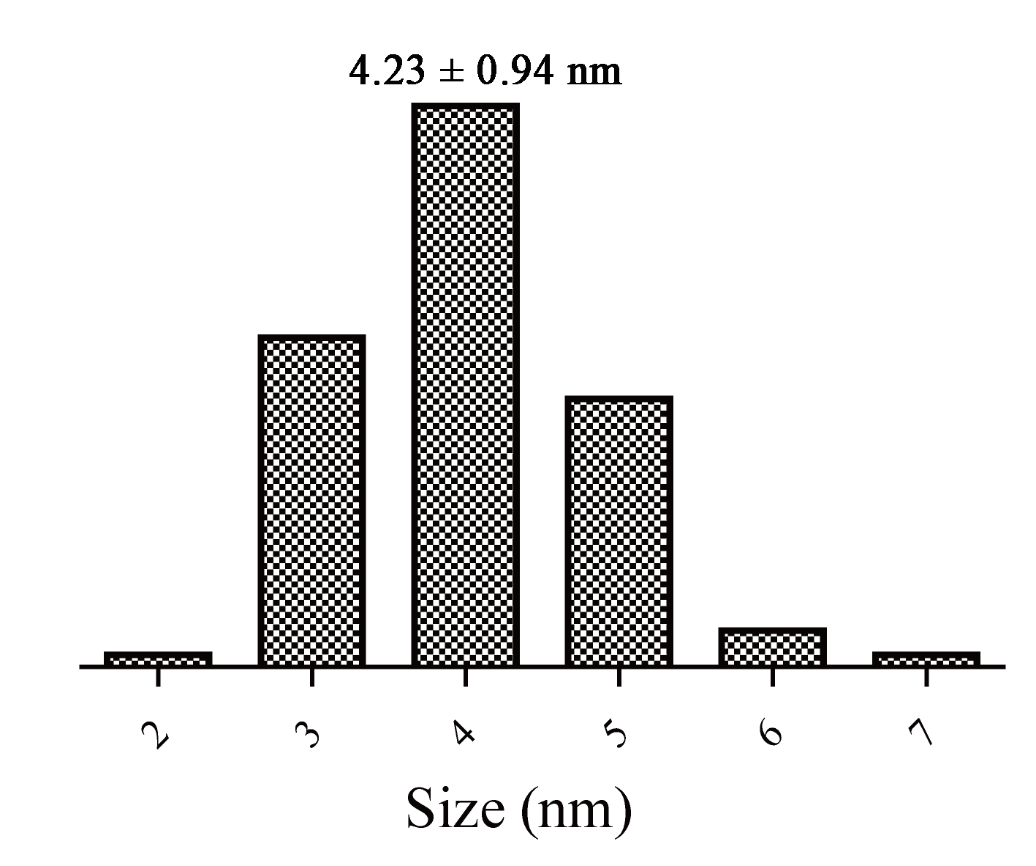


Figure S1. Size distribution of graphene quantum dots. The mean size of GQDs were 4.23 nm, and obtained from corresponding TEM image (n=100).


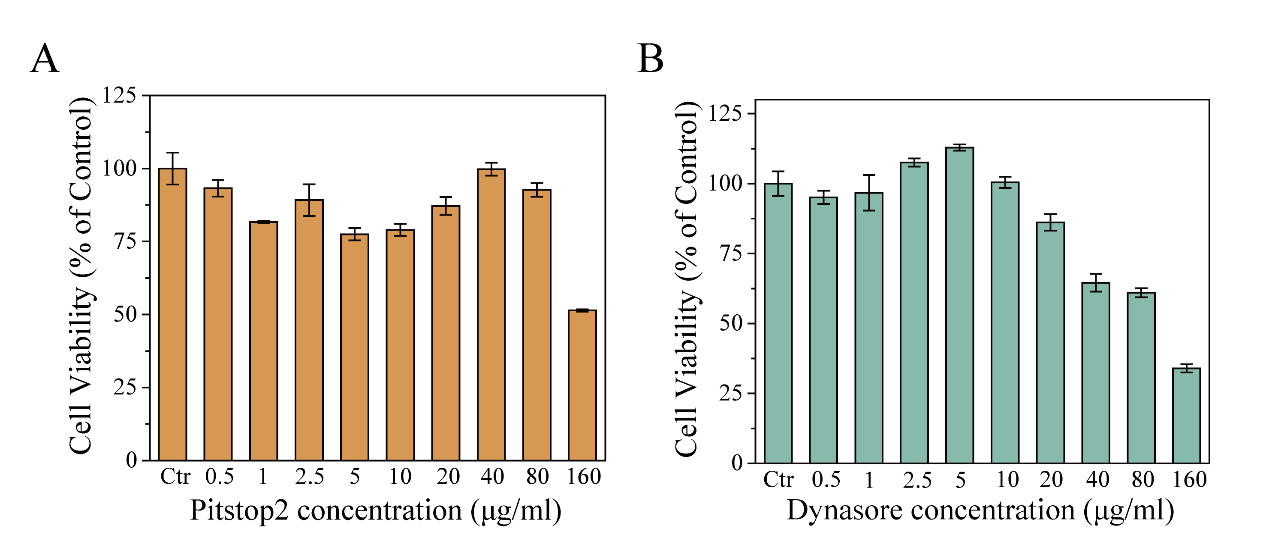


Figure S2. Cell viability of *T. brucei* treated with clathrin inhibitors using CCK-8. (A) *T. brucei* were treated with different concentrations of dynaosre for 3 h. (B) *T. brucei* were treated with different concentrations of pitstop2 for 3 h. Data are shown as mean and standard deviation (n=3).


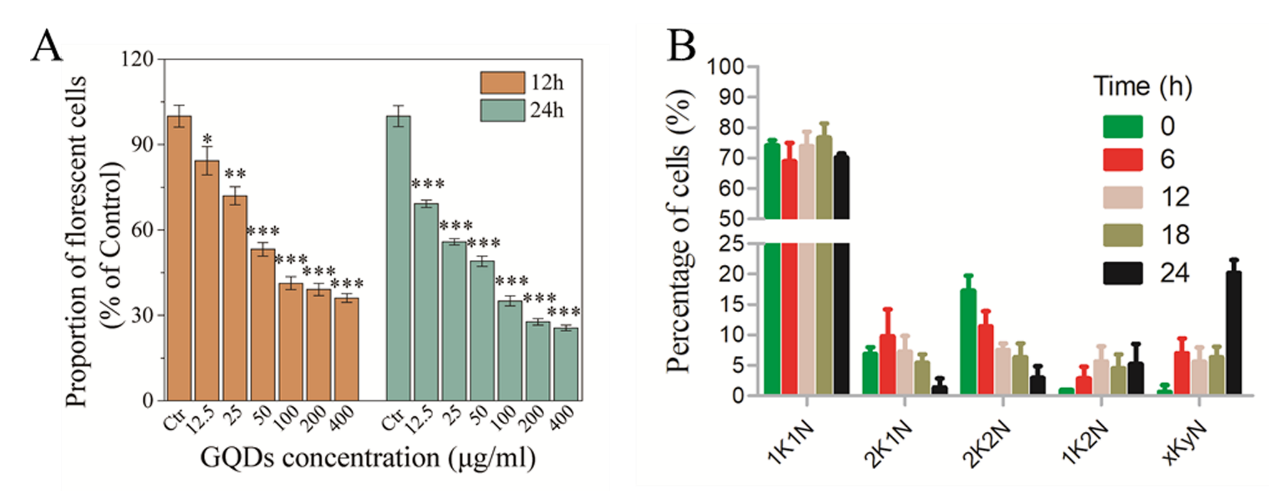


Figure S3. (A) Proportion of florescent *T. brucei* (viable cells) decreased after being treated with a series of concentrations of GQDs detected with PrestoBlue. Results show that *T. brucei* parasites proliferaton decreased with the exposure to increased concentrations of GQDs in 12 and 24 hours. *T. brucei* without GQDs were used as control. (B) Cell morphology phenotypes with nucleus (N) and kinetoplast (K) counts during GQDs (100 μg/ml) treatment within 0-24 h. 1K1N (1 kinetoplast, 1 nucleus), 2K1N, and 2K2N were considered as normal cells.1K2N and xKyN (cells with multiple nuclei and kinetoplast. X and y represents the number of kinetoplast and nuclei) were considered as abnormal cells. K: kinetoplast, N: nucleus. Data are shown as mean and standard deviation (n=3).


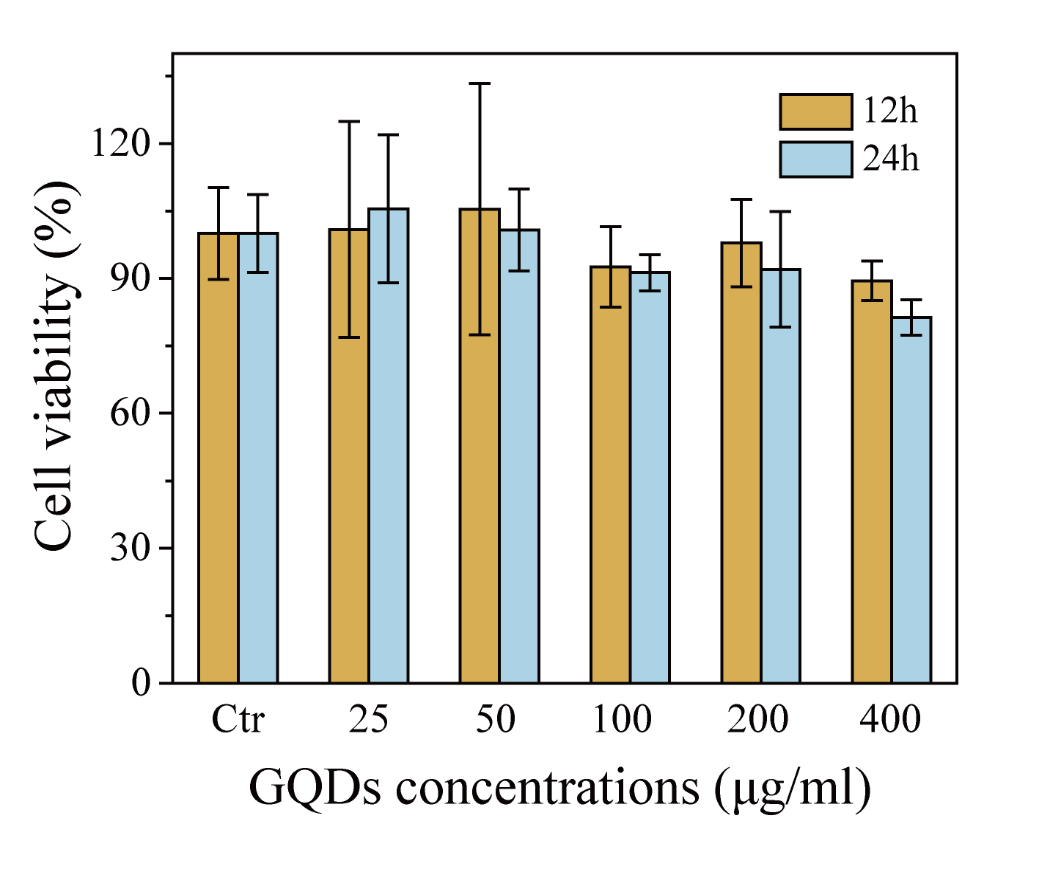


Figure S4. The cell viability assessment of the HepG2 cells after treatment with GQDs. Graphene quantum dots are with hypotoxicity to HepG2 cells.


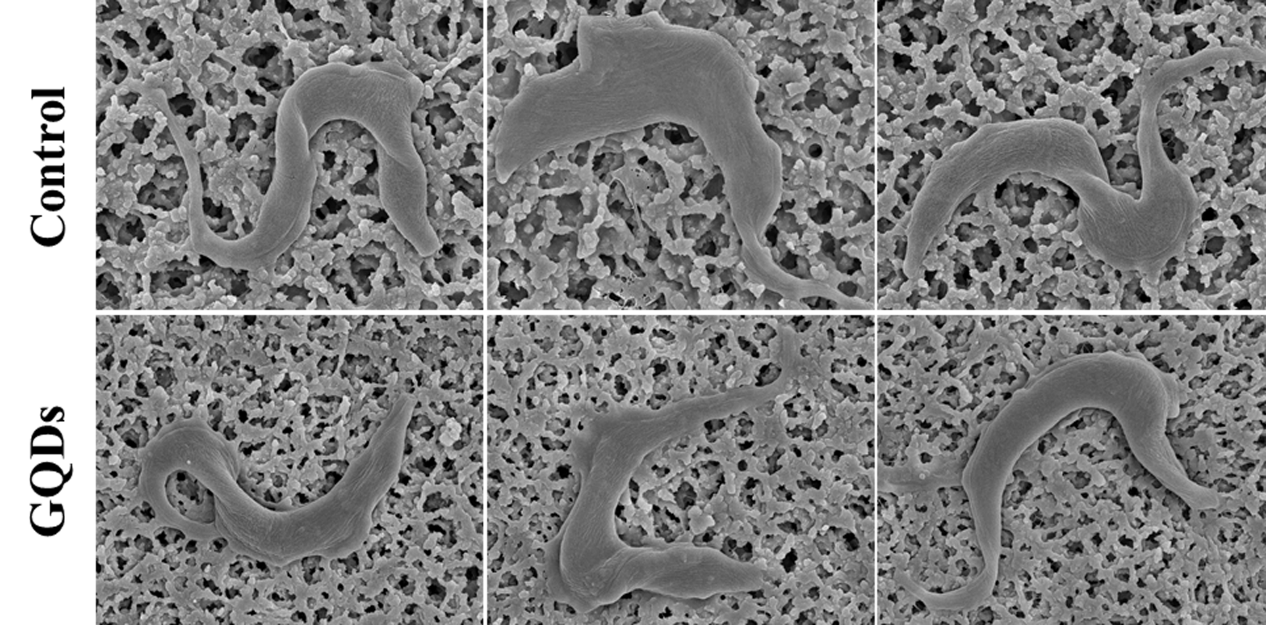


Figure S5. SEM images of *T. brucei* was captured after been cultivated with GQDs (50 μg/ml) for 24 hours. *T. brucei* parasites that were not exposed to GQDs provided as controls.


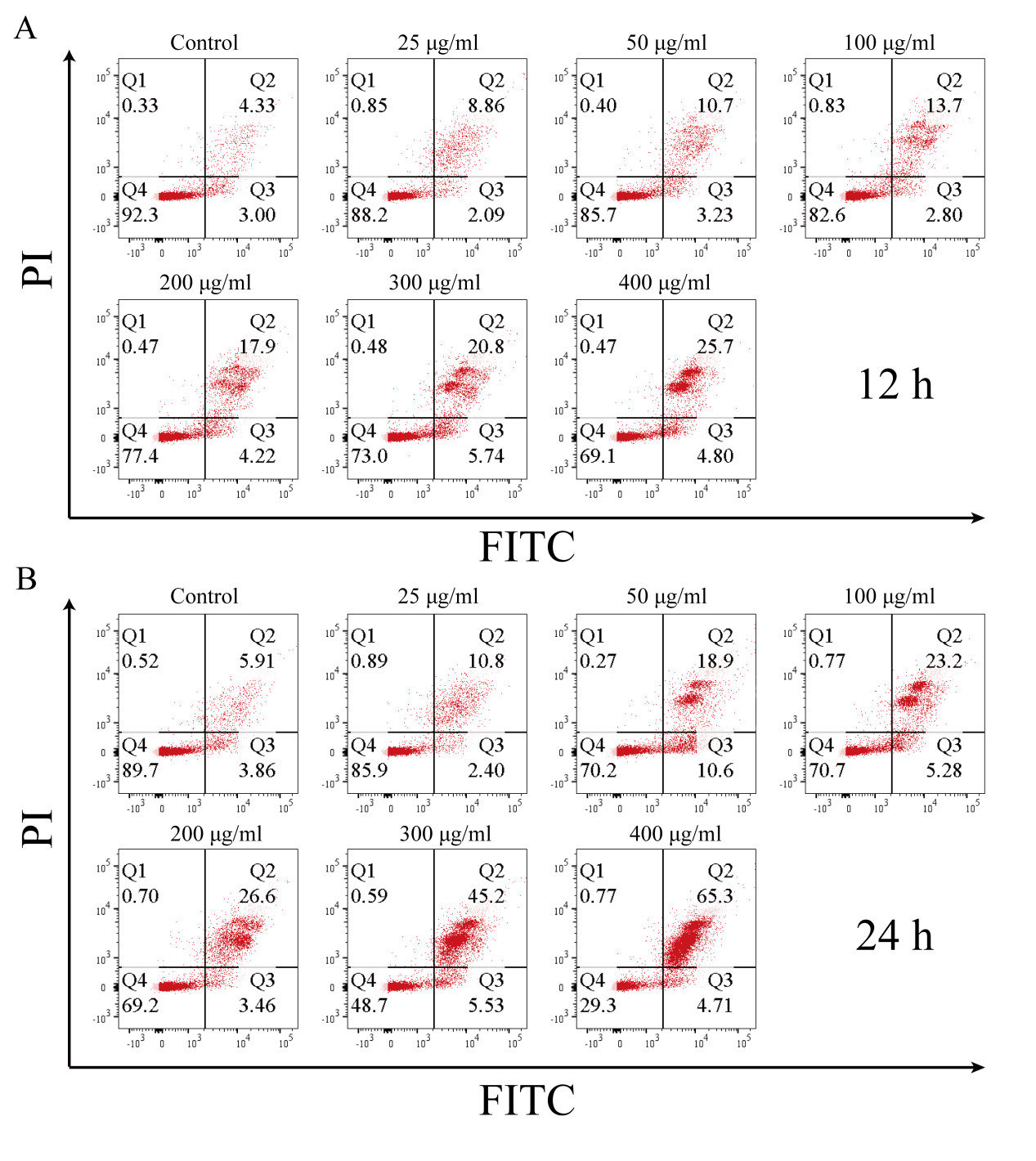


Figure S6. Flow cytometry analysis of apoptosis/necrosis of *T. brucei* after exposure to different concentrations of GQDs for 12 h (A) and 24 h (B), respectively, using Annexin V-FITC/PI apoptosis assay kit. *T. brucei* cells without treatment were used as controls.


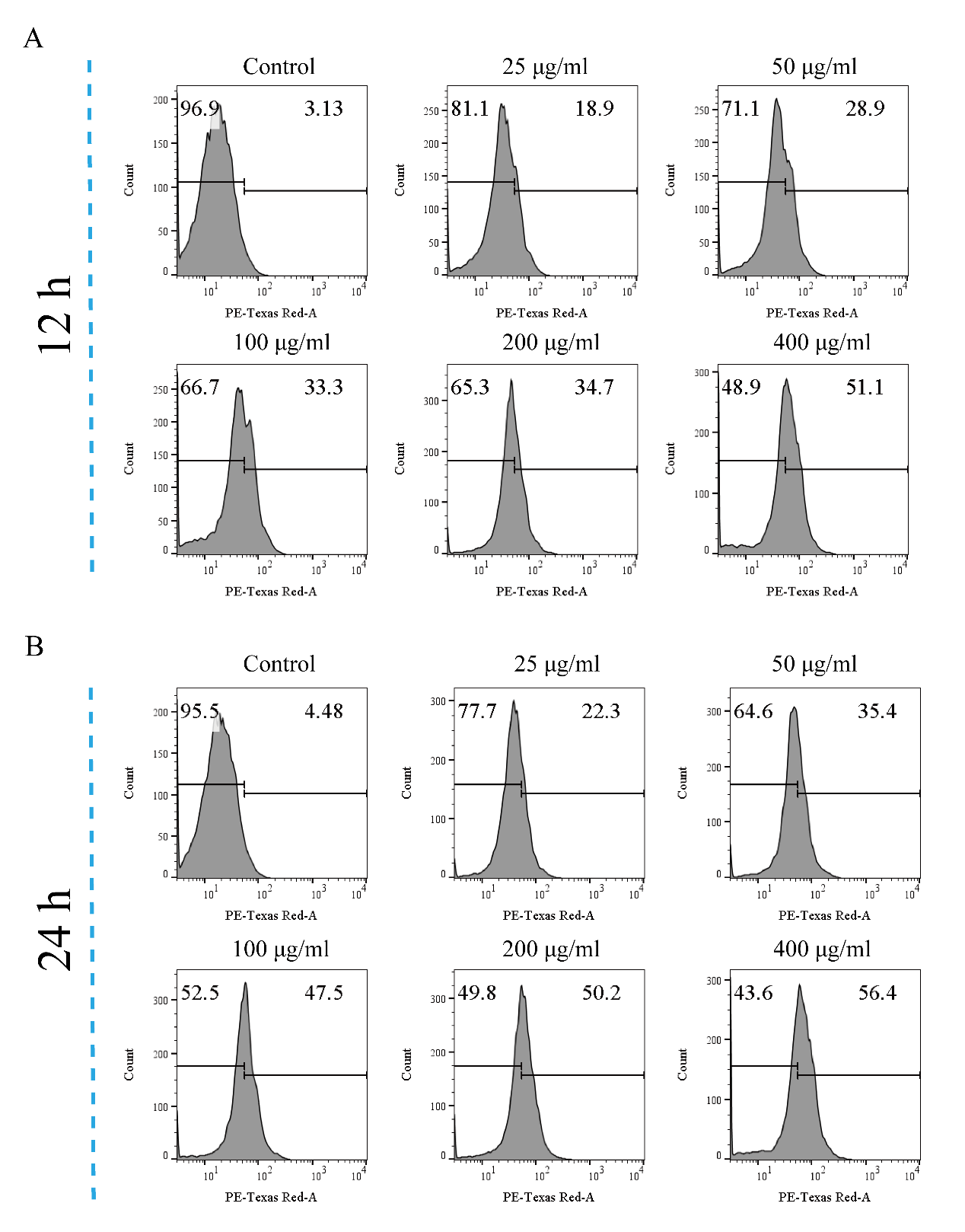


Figure S7. Flow cytometry determination of cellular ROS levels in *T. brucei* with DHE after exposure to the different concentrations of GQDs for 12 h (A) and 24 h (B), respectively.


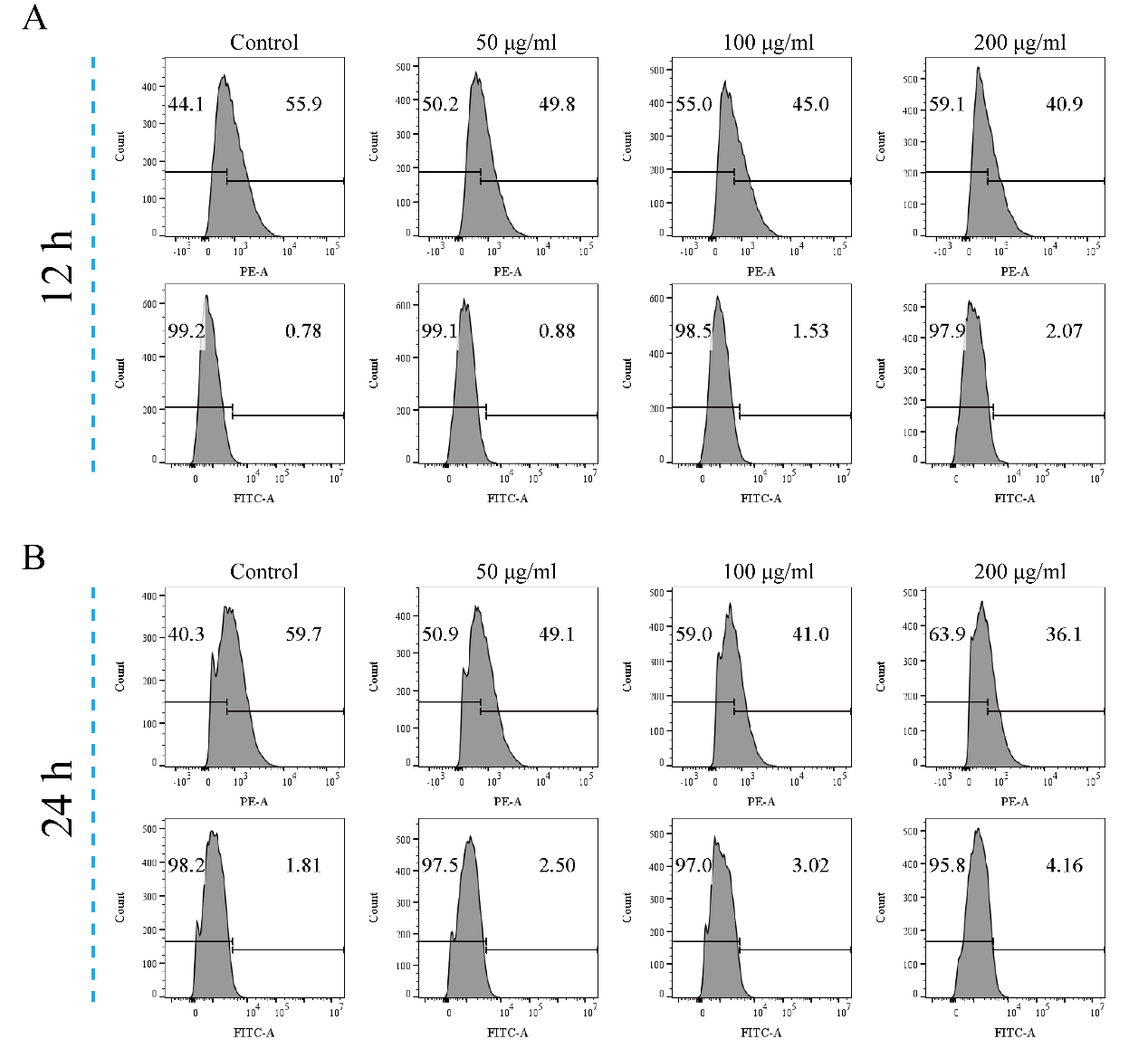


Figure S8. Flow cytometry analysis on mitochondrial membrane potential of *T. brucei* after exposure to different concentrations of GQDs for 12 h (A) and 24 h (B), respectively. *T. brucei* cells without treatment were used as controls.


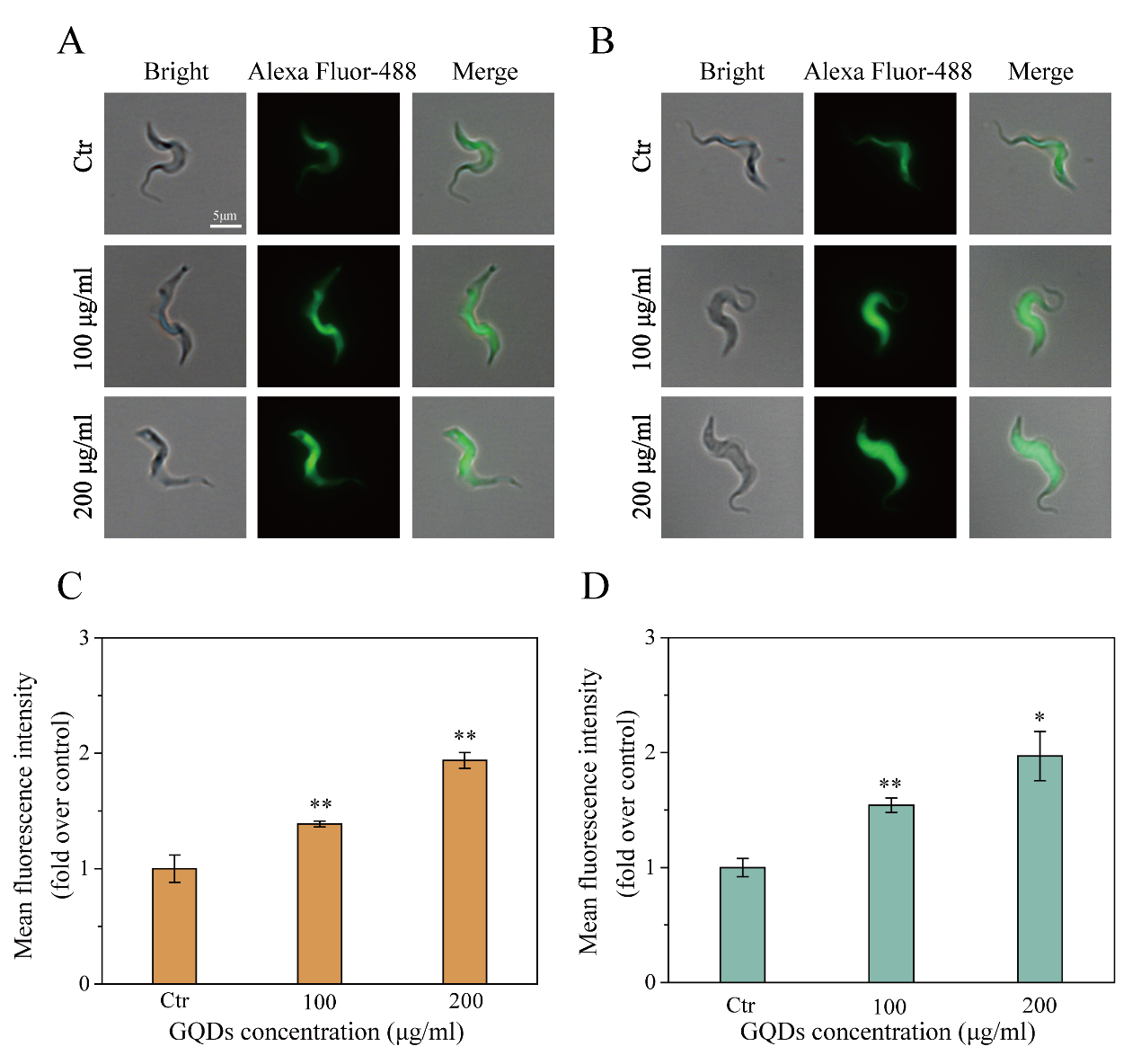


Figure S9. Increased cytoplasmic Ca^2+^ concentration in *T. brucei* detected by laser scanning confocal microscope. (A) *T. brucei* were treated with GQDs for 12 h. (B) *T. brucei* were treated with GQDs 24 h. (C) Densitometric analysis of mean fluorescence intensity images in (A) using ImageJ software (v1.8.0). (D) Densitometric analysis of mean fluorescence intensity images in (B) using ImageJ software (v1.8.0).
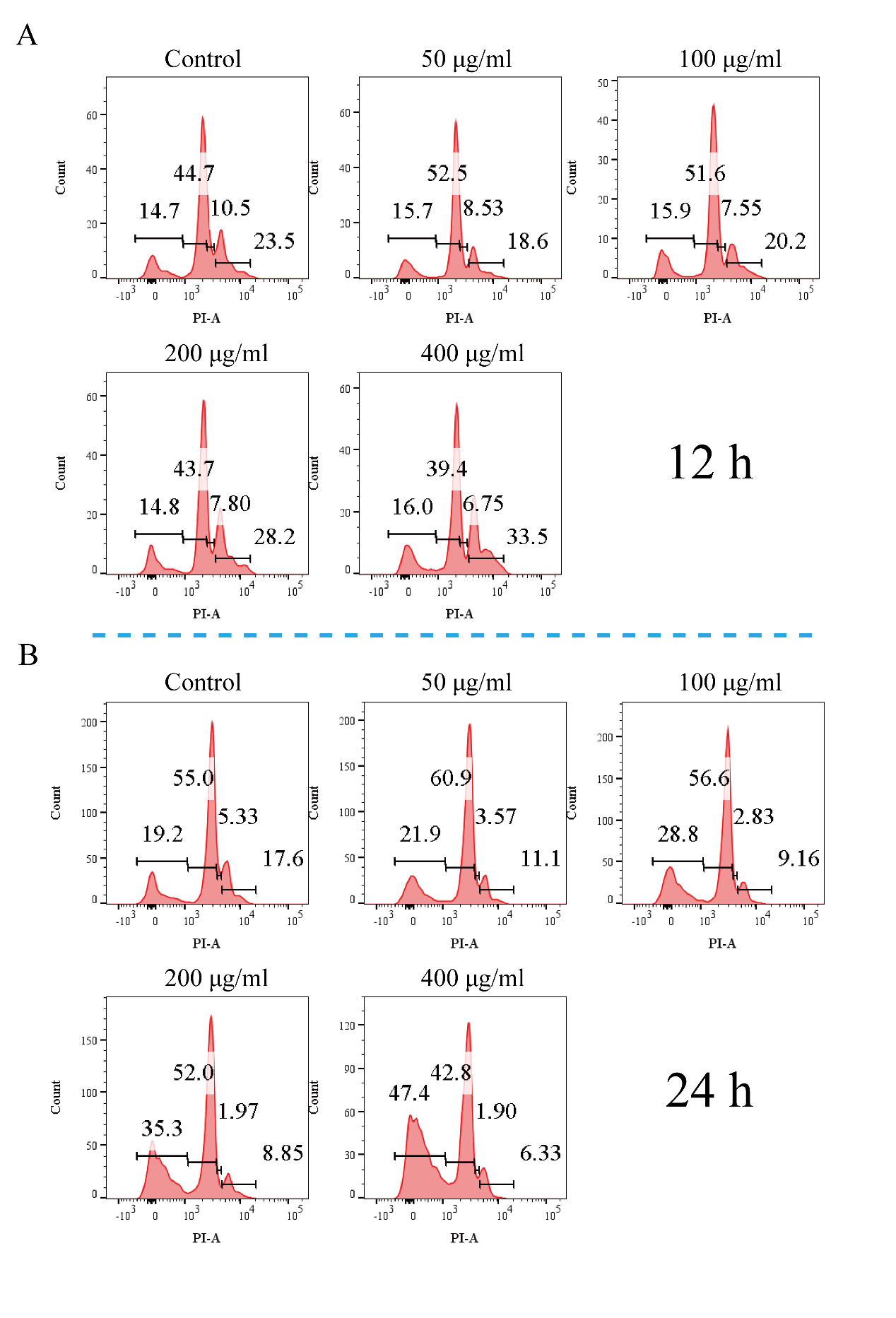


Figure S10. Flow cytometry analysis of cell cycle of *T. brucei* after exposure to GQDs. (A) and (B) *T. brucei* parasites were treated with different concentrations of GQDs for 12 h and 24 h, respectively. *T. brucei* parasites without any treated were used as controls.


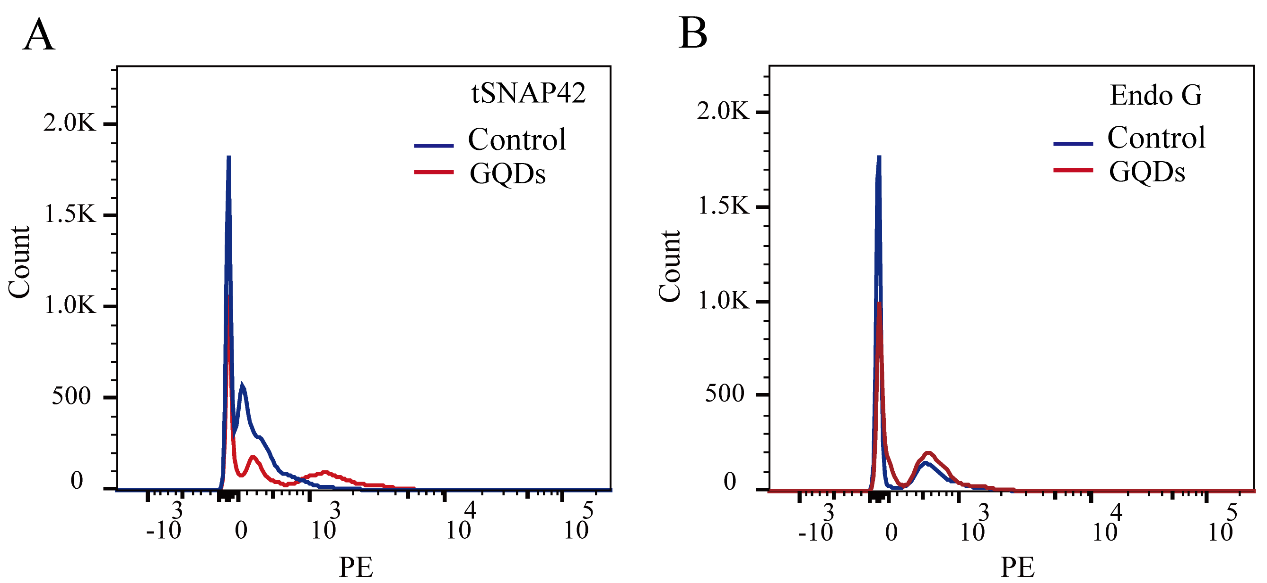


Figure S11. (A) Flow cytometry analysis of intracellular accumulation of tSNAP42 in *T. brucei* after the GQDs treatment. (B) Increased intracellular accumulation of Endo G in *T. brucei* after the GQDs treatment.


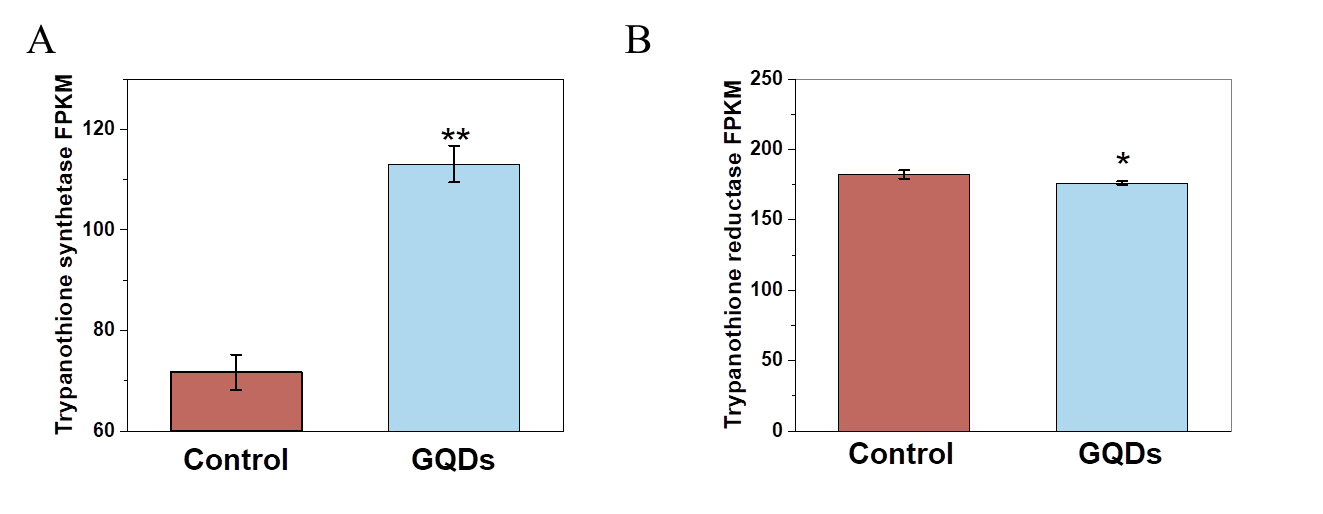


Figure S12. (A) The transcription of trypanothione reductase was decreased after the treatment of GQDs. (B) The transcription of trypanothione synthase was significantly elevated after the treatment of GQDs.


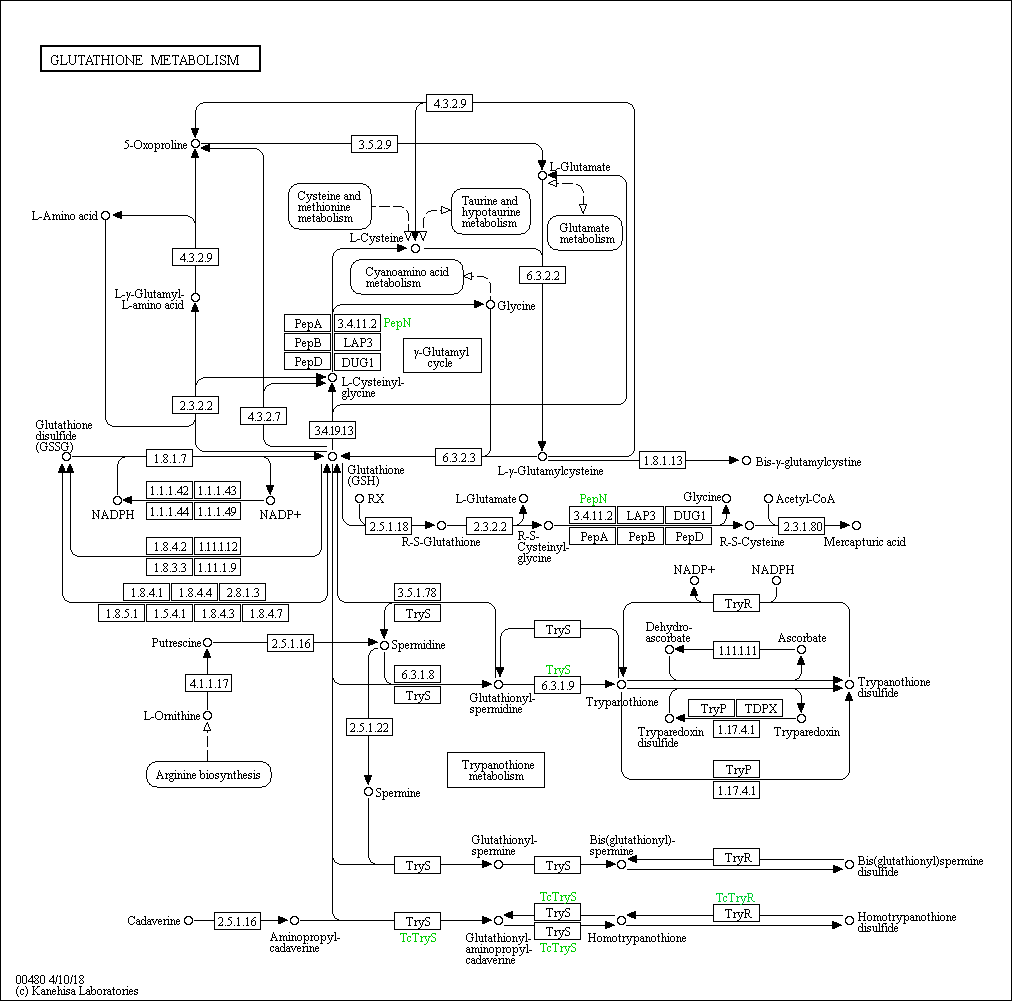


Figure S13. Glutathione metabolism network including trypanothione reductase gene after GQDs treatment. The green represents down-regulated genes.

**2. Supplemental tables**

Table S1. The primer sequences of RT-qPCR

| Gene | Primers |
| --- | --- |
| trypanothione reductase | F: GCAGCCCTTGTGGATACAGTCTTC |
|  | R: GCAGCCCTTGTGGATACAGTCTTC |
| trypanothione synthetase | F:AGTTGTGGATTCGGACGGCATTC |
|  | R: CTTATCCTTTGGCGTGGGCTTCC |
| GADPH | F: AGATTGATGTCGTTGCTGTTGTG |
|  | R: TGCTTGCTCTTCGTAGTCG |

Table S2. Binding kinetics of the Trypanothione reductase to GQDs using

ForteBio system

| Variant | KD(M)^a^ | Kon(1/Ms)^b^ | Kdis(1/s)^c^ | R^2^ |
| --- | --- | --- | --- | --- |
| Tb927.10.10390-His | 5.04E-09 | 2.51E+05 | 1.27E-03 | 0.9701 |
